# Braxon^®^-assisted prepectoral breast reconstruction: A decade later

**DOI:** 10.3389/fsurg.2022.1009356

**Published:** 2022-11-01

**Authors:** Franco Bassetto, Laura Pandis, Federico Facchin, Gian Paolo Azzena, Vincenzo Vindigni

**Affiliations:** Department of Neuroscience: Neurological, Psychiatric, Sensorial, Reconstructive, and Rehabilitative Sciences, University of Padua, Padua, Italy

**Keywords:** acellular dermal matrix, prepectoral breast reconstruction, biocompatibility, Braxon^®^, capsular contracture, cost-effectiveness, radiotherapy, seroma

## Abstract

We are sitting on the cusp of the bioengineered breast era, in which implant-based breast reconstruction is seeing a growing trend and biotechnology research progressively empowers clinical practice. As never before, the choice of biomaterials has acquired great importance for achieving reconstructive outcomes, and the increase in the use of acellular dermal matrices (ADMs) in the field of senology tells us a story of profound upheaval and progress. With the advent of prepectoral breast reconstruction (PPBR), plenty of devices have been proposed to wrap the silicone prosthesis, either completely or partially. However, this has caused a great deal of confusion and dissent with regard to the adoption of feasible reconstructive strategies as well as the original scientific rationale underlying the prepectoral approach. Braxon^®^ is the very first device that made prepectoral implant positioning possible, wrapping around the prosthesis and exerting the proven ADM regenerative potential at the implant–tissue interface, taking advantage of the body's physiological healing mechanisms. To date, the Braxon^®^ method is among the most studied and practiced worldwide, and more than 50 publications confirm the superior performance of the device in the most varied clinical scenarios. However, a comprehensive record of the working of this pioneering device is still missing. Therefore, our aim with this review is to lay a structured knowledge of surgery with BRAXON^®^ and to provide a decision-making tool in the field of PPBR through a complete understanding on the very first device for prepectoral, one decade after its introduction.

## Introduction

In a world increasingly demanding quick and cost-effective medical care strategies, the evolution of techniques and the advancement of biomaterials have raised a global interest in the areas of health management and quality-of-life safekeeping (1).

Breast cancer care and treatment is a prime example of how research and progress have supported high life-enhancing standards for oncological patients (1, 2). Until 60 years ago, while therapeutic approaches were extremely aggressive, causing huge chest wall defects and significant scarring, breast reconstruction was a subject of great controversy, which was regarded as a worthless and risky procedure by highly respected physicians (2, 3). Today, psychophysical impairment caused by such a demolitive surgery is of primary concern, especially considering the global dimension of breast cancer (2, 4). With a progressively aging population and improved diagnostic tools, more than two million women worldwide are diagnosed with breast malignancies every year, thus representing the most frequent cause of female cancer death (1, 5). Driven by such high incidences, medical–surgical research has evolved toward optimized management of breast cancer patients, resulting in less aggressive surgeries and enhanced direct-to-implant (DTI) procedures (6, 7). The establishment of the less invasive skin-sparing mastectomy and nipple-sparing mastectomy approaches, for example, has broadened the scope and viability of different techniques, either prosthetic or autologous type (8). In such a scenario, even if autologous procedures remain excellent reconstructive strategies, their very long operating times, the need for donor site availability, and the associated possible complications explain why many surgeons opt for the simpler and faster implant-based reconstruction (IBR), currently the most common approach (4, 9, 10). In this regard, although improvements in silicone implant safety and fat grafting techniques have been contributing to the growth of prosthetic reconstruction (6, 11), the historical development of IBR has been accompanied first by the introduction and then by the evolution of several generations of collagen-based materials such as acellular dermal matrices (ADMs) (12, 13).

ADMs are human, bovine, or porcine biotechnologically engineered tissue derivatives that are processed (decellularization processes) to remove immunogenic cellular components, while maintaining the structural matrix that encourages angiogenesis and tissue regeneration (12). These collagen biomaterials have generally served a myriad of purposes over time across surgical specialties, resulting particularly in revolutionary breast surgery, where different matrix generations can be identified (12).

After the first application of ADMs in breast surgery was reported in 2001 (14), the earliest human ADM generation became a cornerstone of IBR, with the innovative dual-plane technique initially described by Breuing and Warren. With their experience recorded and published in 2005, breast reconstruction became the immediate and preferred treatment over fully submuscular and delayed reconstructions, thanks to its mitigated psychosocial morbidity, decreased cost, and improved esthetics (9).

Given the effectiveness of this application, a second generation of ADMs continued the dual-plane approach, and they can be identified as xenogenic non-breast-specific ADMs, comprising all those non-human collagen-based materials such as Strattice^®^ that were borrowed from other surgeries for use in the breast (e.g., from abdominal surgery) (15, 16). Breast-inapt membranes greatly enhanced the possibilities of ADM-assisted reconstruction, making them more affordable than the use of the expensive human dermis. However, not being initially indicated and designed for implantation in the breast, their physiochemical properties were not refined, and many postoperative complications emerged in relation to the use of such materials (17).

A real turning point in the application of this type of membrane came with its use in the reaffirmation of breast reconstruction in the prepectoral space, abandoned in the 1980s because of the occurrence of frequent subcutaneous fibrosis due to rejection of the synthetic implant (7, 18). Unlike synthetic materials, ADMs are scientifically proven bioactive scaffolds capable of actively guiding the host response toward a non-inflammatory cellular ingrowth of the implanted biomaterial (19–22). The resulting ADM-promoted regeneration and the newly created tissue prevent fibrosis, substantially reducing the incidence of capsular contracture (CC) (16, 23, 24).

Taking advantage of these regenerative properties, in 2012, Braxon^®^ was patented, the first preshaped biological matrix to completely envelop the breast implant (25). The total biological interface of the implant with the patient’s tissues prevents the onset of fibrotic responses, thus opening the doors to a feasible muscle-sparing technique that allows subcutaneous positioning (25). Starting with Berna et al., more than 50 scientific publications give evidence of the clinical efficacy of this method, making Braxon^®^ one of the most studied breast reconstruction devices in the world. This leap toward breast-specific ADMs defines a third generation of matrices especially tailored to the characteristics of the implantation site, and optimized for prepectoral breast reconstruction (PPBR), giving this technique the possibility to become, in the future, the gold standard in IBR (25–27). As a matter of fact, a large number of studies have demonstrated the feasibility, safety, and advantages of this approach, which today represents the easiest way to reconstruct the breast with an implant, as it replaces the missing volume exactly from where it was removed (26, 28).

To date, a huge variety of synthetic and biological devices have been proposed for use in one-stage PPBR (29). While this great availability of medical devices enriches the range of applicable surgical techniques, it has also led to a great deal of confusion and heterogeneous data that often cloud the choice of the most suitable reconstructive tools. What is currently missing is a high-quality standardized evaluation that produces a homogeneous overview of comparable outcomes.

The purpose of this review is precisely to provide an organized analysis of the main evidence in the field of prepectoral reconstruction, focusing on the biological matrix with the highest number of scientific studies, and this is used with a standardized technique, Braxon^®^ ADM, in order to support increasingly individualized and evidence-based therapies for cancer patients. Here, we report the results of this medical device in the special settings of radiotherapy (RT), revision surgery, and multicenter studies, with a particular focus on seroma and capsular contracture complications, as well as the cost–benefit ratio and reported level of biocompatibility of the biomaterial.

## Methods

As the purpose of this publication is to review the Braxon^®^ standardized PPBR technique, literature research was conducted using only the keyword “Braxon” and selecting studies published between 2014, the year of the first publication, and 2022. All relevant articles were collected using PubMed (https://pubmed.ncbi.nlm.nih.gov/) and Google Scholar (https://scholar.google.it/) databases. A total of 428 scientific articles were collected. Some selection criteria were applied, which are as follows:

1) Only full articles were considered, while abstracts were disregarded;
2) Duplicates were deleted manually; and
3) Articles in which the Braxon^®^ medical device had not been studied or discussed were not taken into consideration.

Following these screening steps, 376 articles were excluded as they did not fit the selection criteria. Overall, 52 publications were identified.

The process of selecting the articles identified in PubMed and Google Scholar can be summarized as follows: 
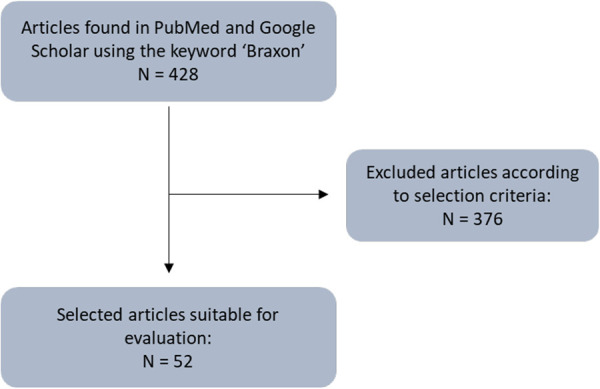


### Surgical technique

**Figure 1:**
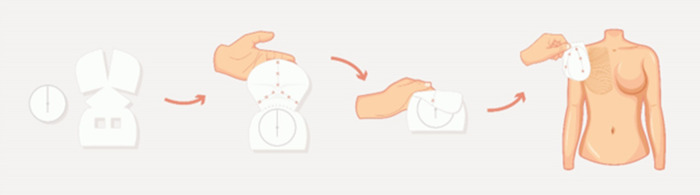
Schematic representation of a Braxon^®^ preshaped design and of the prepectoral technique. Figure adapted from Berna et al., 2014.

Braxon^®^ is a porcine ADM made of natural collagen. It presents as a flat, preshaped collagen sheet with a unique design that allows a complete and continuous wrap of breast prosthesis, either round or anatomical (Figure 1). The standard approach for Braxon^®^ PPBR was first described by Berna et al. using implants ranging from 150 to 450 cc (25). Before use, the ADM must be hydrated for approximately 5 min in a sterile saline solution. After hydration, the matrix becomes pliable and can easily be adapted to the implant. The ADM edges must be sutured together, as shown in Figure 1, tightly wrapping the breast implant and avoiding any bulky seam. The excess of matrix can be removed. Now, the ADM-wrapped implant can be introduced into the breast pocket using parachuting sutures, and the symmetry is verified with the patient in a semiupright position. The Braxon^®^-covered implant is then fixed with apical, medial, and lateral absorbable stitches directly over the pectoralis major muscle. Vacuum drains are then positioned at the inframammary fold (IMF) and also in the axilla if lymph node dissection is performed. The skin is then sutured in two layers after the excision of wound edges. The authors describe the administration of prophylactic antibiotics until the drains are removed 7–15 days after surgery. Women are routinely discharged from hospital with their drains still *in situ* (25). According to the manufacturer's instructions, Braxon^®^ can accommodate implants up to 550 cc; however, there are reports of even bigger implants (585 cc) being used with ADMs (30).

In later publications, other authors have added useful details on the operative technique. They recommend changing gloves before handling the matrix so as to avoid contamination (31). An additional important procedure consists in stitching the anterior side of the dermal matrix to the subcutaneous tissue with quilting sutures using absorbable 2–0 or 3–0 Vicryl stitches (32). Multiple benefits derive from this procedure: it reduces dead spaces and consequently decreases the risk of fluid collection, thereby preventing seroma formation; it ensures intimate contact of the matrix with viable subcutaneous tissues, allowing proper matrix integration and tissue regeneration; it guarantees the mechanical stillness of the implant and prevents frictions that can cause inflammation and hinder matrix integration (32, 33). An additional tip provided by many authors is prescribing the patient to wear a sport bra starting from 2 days after surgery and for at least 1 month (34). As regards drains, they should be removed when the collected fluid remains in the range of 20–30 cc for at least two consecutive days (26, 32, 35, 36).

Braxon^®^-assisted PPBR is indicated in cases of skin- and nipple-sparing mastectomy (25). However, there is evidence of Braxon^®^ usage following skin-reducing mastectomy, with a modified surgical technique. In 2019, Onesti et al. first described this procedure. Braxon^®^-covered implants were placed above the pectoralis muscle and covered inferiorly by a dermal flap. The ADM was sutured with lateral, apical, and medial resorbable sutures to the anterior fascia of the pectoralis major. To define the IMF, the pocket was closed laterally attaching the dermal flap to the fascia of the pectoralis muscle and of the serratus anterior. The upper edge was sutured to the ADM, mimicking a natural bra. The skin was closed in the inverted-T fashion after a drain was placed into the pocket. When possible, the nipple–areola complex was harvested as a full-thickness skin graft and grafted back in the new position (37). The same technique has been used by other surgeons (35, 38, 39). In particular, Maruccia et al. also performed quilting sutures between the ADM and the dermal flap (35).

Limited evidence exists about the use of Braxon® with expanders. Such use is reported in only one publication in which authors adapted the DTI procedure and implanted an expander into patients whose nipple had to be sacrificed. (40).

In 2020, Cuomo et al. presented a study to show the results of intraoperative procedures performed with the aim of improving the esthetic results of prepectoral reconstructions. Initially, the surgeon would create a croissant-shaped mark with a downward concavity in the proximal part of the upper pole of the breast on patients undergoing nipple- or skin-sparing mastectomy. During the surgical procedure, the gland is detached from the subcutaneous fat, leaving approximately 1 cm of tissue but saving approximately 2 cm of subcutaneous fat in the area with the croissant-like mark to improve the fullness of the upper extremity of the superior pole. The Braxon®-wrapped prosthesis is then implanted as previously described. With this modified technique, breast contour and upper pole definition proved to be better than the traditional procedure in 14 operated patients. It should be noted that such results can be obtained when patients meet some specific criteria, which are as follows: cancer localization in the inferior pole, no planned postsurgery RT, an estimated breast volume <500 cm^3^, and an abundance of fat on the upper pole (41).

The Braxon® technique has been described in revision surgery as well (30, 42, 43). In the case of revision for treating capsular contracture, prior implant positioning, the old capsule is removed anteriorly to allow the integration of the Braxon ADM with the refreshed tissue of the pocket (30). In the case of plane conversion, the pectoralis muscle is dissected from the subcutaneous tissue and repositioned onto the chest wall (43).

The breast subcutaneous tissue is the key player in PPBR because it must provide the cells that will repopulate the ADM and use it as a regenerative scaffold. Proper matrix integration is an essential step in limiting postoperative complications (33, 44, 45). Hereby, it is extremely important to preserve tissue viability and vascularization, avoiding, where possible, all those intraoperative procedures during mastectomy and reconstruction that could impair it. The surgical technique for Braxon® implantation can vary depending on the application. Nevertheless, there are fundamental passages and surgical tips, listed in Table 1, that are valid for all applications. Additional preoperative, intraoperative, and postoperative measures can be found in the article published by Knight et al., 2020 (31).

**Table 1:** Braxon® surgical steps.

Mastectomy	Reconstruction
• Reduce the use of a monopolar electrocautery (or electric scalpel) in favor of the cold scalpel during mastectomy or use it at a lower temperature/power. • If lymph node dissection is to be performed, the creation of two separate chambers (axilla and breast) is recommended. • Avoid the use of povidone-iodine for rinsing the breast pocket (impairs tissue viability). • Avoid the use of harsh retractors as they may cause necrotic areas on the skin. • Spare as much subcutaneous tissue as possible to foster greater blood supply to the matrix.	• Braxon® hydration for 5 min in room temperature saline solution. • No rinsing of Braxon® in povidone-iodine. If done for prosthesis, rinse thoroughly. • Create a snug wrapping of the implant with interrupted adsorbable stitches, beginning with the flaps that will create the anterior dome. When the anterior and the posterior flap are sutured together, cut the matrix excess. • Insert the Braxon®-wrapped implant using parachuting sutures to fix it to the farthest point from the incision. • Fix the Braxon® envelope above the pectoralis major muscle with multiple stitches placed at cardinal points. • Fix the matrix with quilting sutures to the subcutaneous tissue to ensure primary stability, obliteration of dead spaces, and the closest possible contact with living tissue. Fibrin glue can be used as an alternative. • Place separate drains for the breast and for the axilla. Drains should be removed when the collected fluid is 20–30 cc for 48 h. • Perform excision of wound edges before closing the wound. • The patient should wear a surgical supportive bra (or sport bra) for at least 15 days so that the breast remains as stable as possible.

As for every device introduced in clinical practice, there is also a learning curve associated with Braxon®. After familiarizing oneself with the ADM and the technique, one will find PPBR to be a simple and standardized procedure that offers oncological patients a less invasive reconstructive strategy, while at the same time increasing the satisfaction levels of surgeons. Deviating from the correct practice and not respecting the surgical indication, especially during the learning curve, leads to a higher risk of postoperative complications (40). In addition, optimal reconstructive outcomes strictly depend on correct patient selection, as discussed in the next paragraph.

For a comprehensive list of the Braxon®-specific articles cited in this paragraph, see Table 2.

**Table 2:** List of the Braxon®-specific published articles subdivided by topics.

Topics	Authors	Publication
Surgical technique	Berna et al., 2014 (25)	Evaluation of a novel breast reconstruction technique using the Braxon® acellular dermal matrix: a new muscle-sparing breast reconstruction.
	Vidya et al., 2017 (26)	Evaluation of the effectiveness of the prepectoral breast reconstruction with Braxon dermal matrix: first multicenter European report on 100 cases.
	Onesti et al., 2017 (45)	Clinical, histological, and ultrasound follow-up of breast reconstruction with one-stage muscle-sparing “wrap” technique: a single-center experience.
	Vidya, 2017 (36)	Prepectoral breast reconstruction or muscle-sparing technique with the Braxon porcine acellular dermal matrix
	Vidya et al., 2017 (32)	Muscle-sparing ADM-assisted breast reconstruction technique using complete breast implant coverage: a dual-institute UK-based experience.
	Kankam et al., 2018 (38)	Combination of acellular dermal matrix with a de-epithelialised dermal flap during skin-reducing mastectomy and immediate breast reconstruction.
	Onesti et al., 2019 (37)	ADM-assisted prepectoral breast reconstruction and skin reduction mastectomy: expanding the indications for subcutaneous reconstruction.
	Mangialardi et al., 2019 (43)	Delayed acellular dermal matrix assisted prepectoral breast reconstruction: preliminary results.
	Knight et al., 2019 (31)	Significantly reducing implant loss rates in immediate implant-based breast reconstruction: a protocol and completed audit of quality assurance.
	Cuomo et al., 2020 (41)	Optimization of prepectoral breast reconstruction.
	Maruccia et al., 2020 (35)	Skin-reducing mastectomy and pre-pectoral breast reconstruction in large ptotic breasts.
	Ribuffo et al., 2020 (34)	Dual-plane retro-pectoral versus pre-pectoral DTI breast reconstruction: an Italian multicenter experience.
	Naemonitou et al., 2020 (39)	Outcome of complete acellular dermal matrix wrap with polyurethane implant in immediate prepectoral breast reconstruction.
	Mura et al., 2021 (33)	Direct-to-implant, prepectoral breast reconstruction with Braxon® dermal matrix: a single-center experience with 111 cases.
	Caputo et al., 2021 (44)	Seroma formation in pre-pectoral implant-based adm assisted breast reconstruction: a comprehensive review of current literature.
	Bojanic et al., 2021 (40)	Indications and pitfalls of prepectoral breast reconstruction with Braxon® Acellular Dermal Matrix (ADM): a preliminary plastic surgical experience.
	Bojanic et al., 2021 (42)	First use of Braxon® acellular dermal matrix for complex revision aesthetic breast surgery—revision augmentation mastopexy.
	Bassetto et al., 2022 (30)	Complete implant wrapping with porcine-derived acellular dermal matrix for the treatment of capsular contracture in breast reconstruction: a case–control study.
Patient selection and preoperative management	Berna et al., 2014 (25)	Evaluation of a novel breast reconstruction technique using the Braxon® acellular dermal matrix: a new muscle-sparing breast reconstruction.
	Maruccia et al., 2016 (46)	One-stage breast reconstruction techniques in elderly patients to preserve quality of life.
	Onesti et al., 2017 (45)	Clinical, histological, and ultrasound follow-up of breast reconstruction with one-stage muscle-sparing “wrap” technique: a single-center experience.
	Vidya et al., 2017 (29)	A guide to prepectoral breast reconstruction: a new dimension to implant-based breast reconstruction.
	Vidya et al., 2018 (47)	Management based on grading of animation deformity following implant-based subpectoral breast reconstruction.
	Vidya et al., 2019 (48)	Minimal pain with prepectoral implant-based breast reconstruction.
	Vidya et al., 2019 (49)	Prepectoral implant-based breast reconstruction: a joint consensus guide from UK, European and USA breast and plastic reconstructive surgeons.
	Vidya et al., 2019 (50)	Rippling associated with pre-pectoral implant based breast reconstruction: a new grading system.
	Mangialardi et al., 2019 (43)	Delayed acellular dermal matrix assisted prepectoral breast reconstruction: preliminary results.
	Maruccia et al., 2020 (35)	Skin-reducing mastectomy and pre-pectoral breast reconstruction in large ptotic breasts.
	Polotto et al., 2020 (51)	One-step prepectoral breast reconstruction with porcine dermal matrix-covered implant: a protective technique improving the outcome in post-mastectomy radiation therapy setting.
	Mangialardi et al., 2020 (52)	Prepectoral implant pocket conversion in breast reconstruction.
	Masià et al., 2020 (53)	The largest multicentre data collection on prepectoral breast reconstruction: The iBAG study.
	Mura et al., 2021 (33)	Direct-to-implant, prepectoral breast reconstruction with Braxon® dermal matrix: a single-center experience with 111 cases.
	Bassetto et al., 2022 (30)	Complete implant wrapping with porcine-derived acellular dermal matrix for the treatment of capsular contracture in breast reconstruction: a case–control study.
	Saibene et al., 2022 (27)	Incidence of capsular contracture on irradiated acellular dermal matrices (ADMs)-assisted prepectoral breast reconstructions.
Capsular contracture	Berna et al., 2014 (25)	Evaluation of a novel breast reconstruction technique using the Braxon® acellular dermal matrix: a new muscle-sparing breast reconstruction.
	Maruccia et al., 2016 (46)	One-stage breast reconstruction techniques in elderly patients to preserve quality of life.
	Berna and Cawthorn., 2017 (54)	Long term follow-up on prepectoral ADM-assisted breast reconstruction: evidences after 4 years.
	Onesti et al., 2017 (45)	Clinical, histological, and ultrasound follow-up of breast reconstruction with one-stage muscle-sparing “wrap” technique: a single-center experience.
	Chandarana et al., 2018 (55)	Acellular dermal matrix in implant-based immediate breast reconstructions: a comparison of prepectoral and subpectoral approach.
	Gardani et al., 2018 (56)	Prepectoral breast reconstruction using the Braxon® porcine acellular dermal matrix: a retrospective study.
	Chandarana et al., 2019 (57)	Outcomes of prepectoral implant-based breast reconstruction with Braxon® acellular dermal matrix—a single-center experience.
	Ballesio et al., 2019 (58)	Postsurgical ultrasound evaluation of patients with prosthesis in Acellular Dermal Matrix: results from monocentric experience.
	Onesti et al., 2020 (37)	ADM-assisted prepectoral breast reconstruction and skin reduction mastectomy: expanding the indications for subcutaneous reconstruction.
	Chandarana et al., 2020 (59)	Multicentre study of prepectoral breast reconstruction using acellular dermal matrix.
	Polotto et al., 2020 (51)	One-step prepectoral breast reconstruction with porcine dermal matrix-covered implant: a protective technique improving the outcome in post-mastectomy radiation therapy setting.
	Maruccia et al., 2020 (35)	Skin-reducing mastectomy and pre-pectoral breast reconstruction in large ptotic breasts.
	Naemonitou et al., 2020 (39)	Outcome of complete acellular dermal matrix wrap with polyurethane implant in immediate prepectoral breast reconstruction.
	Spengler et al., 2021 (60)	Lessons learned from three different acellular dermal matrices in direct-to-implant breast reconstruction.
	Mura et al., 2021 (33)	Direct-to-implant, prepectoral breast reconstruction with Braxon® dermal matrix: a single-center experience with 111 cases.
	Bojanic et al., 2021 (40)	Indications and pitfalls of prepectoral breast reconstruction with Braxon® Acellular Dermal Matrix (ADM): a preliminary plastic surgical experience.
	Maruccia et al., 2021 (61)	Prepectoral breast reconstruction: an ideal approach to bilateral risk-reducing mastectomy.
	Gardani et al., 2022 (62)	Skin-reducing mastectomy and prepectoral breast reconstruction using the Braxon ® ADM: a single-centre experience.
	Saibene et al., 2022 (27)	Incidence of capsular contracture on irradiated acellular dermal matrices (ADMs)-assisted prepectoral breast reconstructions.
Biocompatibility and adipogenic potential	Iqbal et al., 2016 (63)	Host integration of an acellular dermal matrix: Braxon mesh in breast reconstruction.
	Onesti et al., 2017 (45)	Clinical, histological, and ultrasound follow-up of breast reconstruction with one-stage muscle-sparing “wrap” technique: a single-center experience.
	Ballesio et al., 2019 (58)	Postsurgical ultrasound evaluation of patients with prosthesis in acellular dermal matrix: results from monocentric experience.
	Quintero et al., 2022 (64)	Tissue-material integration and biostimulation study of collagen acellular matrices.
Seroma	Berna et al., 2014 (25)	Evaluation of a novel breast reconstruction technique using the Braxon® acellular dermal matrix: a new muscle-sparing breast reconstruction.
	Maruccia et al., 2016 (46)	One-stage breast reconstruction techniques in elderly patients to preserve quality of life.
	Jafferbhoy et al., 2017 (65)	Early multicentre experience of pre-pectoral implant based immediate breast reconstruction using Braxon®.
	Onesti et al., 2017 (45)	Clinical, histological, and ultrasound follow-up of breast reconstruction with one-stage muscle-sparing “wrap” technique: a single-center experience.
	Vidya et al., 2017 (26)	Evaluation of the effectiveness of the prepectoral breast reconstruction with Braxon dermal matrix: first multicenter European report on 100 cases.
	Vidya and Cawthorn, 2017 (32)	Muscle-sparing ADM-assisted breast reconstruction technique using complete breast implant coverage: a dual-institute UK-based experience.
	Gardani et al., 2018 (56)	Prepectoral breast reconstruction using the Braxon® porcine acellular dermal matrix: a retrospective study.
	Chandarana et al., 2018 (55)	Acellular dermal matrix in implant-based immediate breast reconstructions: a comparison of prepectoral and subpectoral approach.
	Chandarana et al., 2019 (57)	Outcomes of prepectoral implant-based breast reconstruction with Braxon® acellular dermal matrix—a single-centre experience.
	Ballesio et al., 2019 (58)	Postsurgical ultrasound evaluation of patients with prosthesis in acellular dermal matrix: results from monocentric experience.
	Chandarana et al., 2020 (59)	Multicentre study of prepectoral breast reconstruction using acellular dermal matrix.
	Onesti et al., 2019 (37)	ADM-assisted prepectoral breast reconstruction and skin reduction mastectomy: expanding the indications for subcutaneous reconstruction.
	Masià et al., 2020 (53)	The largest multicenter data collection on prepectoral breast reconstruction: The iBAG study.
	Caputo et al., 2021 (44)	Seroma formation in pre-pectoral implant-based adm assisted breast reconstruction: a comprehensive review of current literature.
Radiotherapy	Polotto et al., 2020 (51)	One-step prepectoral breast reconstruction with porcine dermal matrix-covered implant: a protective technique improving the outcome in post-mastectomy radiation therapy setting.
	Masià et al., 2020 (53)	The largest multicentre data collection on prepectoral breast reconstruction: The iBAG study.
	Saibene et al., 2022 (27)	Incidence of capsular contracture on irradiated acellular dermal matrices (ADMs)-assisted prepectoral breast reconstructions.
Revision surgery	Berna et al., 2014 (25)	Evaluation of a novel breast reconstruction technique using the Braxon® acellular dermal matrix: a new muscle-sparing breast reconstruction.
	Mangialardi et al., 2019 (43)	Delayed acellular dermal matrix assisted prepectoral breast reconstruction: preliminary results.
	Mangialardi et al., 2020 (52)	Prepectoral implant pocket conversion in breast reconstruction.
	Fanelli et al., 2021 (66)	Thoracic migration of silicone gel after breast implant rupture: a case report and literature review.
	Bojanic et al., 2021 (42)	First use of Braxon® acellular dermal matrix for complex revision aesthetic breast surgery—revision augmentation mastopexy.
	Bassetto et al., 2022 (30)	Complete implant wrapping with porcine-derived acellular dermal matrix for the treatment of capsular contracture in breast reconstruction: a case–control study.
Cost-effectiveness	Linguadoca et al., 2017 (67)	L’HTA applicato alla ricostruzione mammaria in interventi chirurgici one-step: dall’utilizzo degli espansori all’impiego di matrici biologiche, l’esperienza dell’Azienda Ospedaliero- Universitaria di Parma.
	Cattelani et al., 2018 (68)	One-step prepectoral breast reconstruction with dermal matrix–covered implant compared to submuscular implantation: functional and cost evaluation.
	Cattelani et al., 2018 (69)	The economics of prepectoral breast reconstruction.
	Innocenti et al., 2022 (70)	Two-stage expander/implant breast reconstruction versus prepectoral breast reconstruction with acellular dermal matrix: a cost analysis.
Quality of life	Maruccia et al., 2016 (46)	One-stage breast reconstruction techniques in elderly patients to preserve quality of life.
	Onesti et al., 2017 (45)	Clinical, histological, and ultrasound follow-up of breast reconstruction with one-stage muscle-sparing “wrap” technique: a single-center experience
	Maruccia et al., 2018 (71)	One-stage muscle-sparing breast reconstruction in elderly patients: a new tool for retaining excellent quality of life.
	Cattelani et al., 2018 (68)	One-step prepectoral breast reconstruction with dermal matrix–covered implant compared to submuscular implantation: functional and cost evaluation.
	Onesti et al., 2019 (37)	ADM-assisted prepectoral breast reconstruction and skin reduction mastectomy: expanding the indications for subcutaneous reconstruction
	Caputo et al., 2020 (72)	Quality of life and early functional evaluation in direct-to-implant breast reconstruction after mastectomy: a comparative study between prepectoral versus dual-plane reconstruction.

Authors, reference numbers, and titles are reported.

### Patient selection and preoperative management

Braxon® has been used in breast-reconstructive, esthetic, and revision surgery (42, 43, 53, 73). For all these applications, meticulous patient selection is of great importance in order to limit postoperative complications and obtain satisfactory, esthetically valuable, and safe results. ADM integration on the prepectoral plane is strongly related to the vitality of the mastectomy flap. In fact, a good subcutaneous layer and well-perfused skin flaps are of paramount importance in favoring Braxon® biological activity and limiting postoperative complications (49, 74).

Most authors suggest performing an ADM-assisted PPBR on patients selected with strict criteria: non- or ex-smokers, non-obese (BMI < 30 kg/m^2^) people with a history of undergoing a considerable number of preoperative pinch tests (>1 cm), without a history of or planned RT, limited implant volume, and no comorbidities, especially if impairing vascularization. In addition to optimal skin quality, only mild to moderately ptotic breasts of small to medium size and subjected to a nipple- or skin-sparing mastectomy are generally considered for DTI PPBR (25, 29, 33, 49, 53). In addition, inflammatory tumors and skin invasion are considered contraindications (49). Additional risk factors such as known hypersensitivity to animal-derived materials or collagen-related pathologies must be considered, as well as patients refusing xeno-derived products (30).

In Table 3, the exclusion criteria for Braxon®-assisted PPBR are reported, which are divided as systemic and local factors.

**Table 3:** Summary of risk factors and exclusion criteria reported in the literature specific for Braxon®-assisted prepectoral breast reconstruction.

Systemic factors	Local factors
Smoking Hx	Hx of RT (pre or post op)
BMI >30	Breast volume >450 cc
Diabetes	Thin/compromised mastectomy flap
Vascular disease	Pinch test <1 cm
Immunodeficiency/immunosuppressive drug use	Ptosis grade III
Animal-derived material hypersensitivity	Axillary dissection
Collagen-related pathologies	Candidates for skin-reducing mastectomy
Psychiatric disorders incompatible with postoperative management	Tumor invading the skin/inflammatory nature of the tumor

Hx, history; BMI, body mass index; RT, radiotherapy; ADM, acellular dermal matrices.

Adapted from Masià et al. (2020).

As for patients undergoing prosthetic revision, the exclusion criteria are usually less restrictive, thanks to the usually good condition of skin flaps (75). Nonetheless, morbid obesity, thin skin, history of RT, and implant volume higher than 400 cc would discourage the use of ADMs (76). A specific additional selection criterion may be applied, depending on the clinical case, preferring a thicker mastectomy flap at the time of taking the pinch test (43, 52).

The aforementioned strict patient selection/exclusion criteria are to be always followed, especially during the early learning curve, although it should be noted that, in these 10 years of Braxon®-assisted PPBR, those criteria were not always followed. However, the silver lining is that Braxon® efficacy could also be studied for treating those who were considered “non-ideal” patients. The positive outcomes of such reconstruction procedures, despite a slight increase in postoperative complications (as could be expected), have led to a broadening of the selection criteria. Now, the Braxon® technique can also be offered, always after evaluating case by case, to patients with comorbidities (yet presenting no more than two), with BMI < 35 kg/m^2^, candidates with a skin-reducing mastectomy, those who have taken a pinch test <1 cm (yet having good tissue vascularization), and who are receiving postoperative RT (27, 35, 45, 46, 51, 53).

Patient risk factors are taken into consideration during the preoperative planning stage, with the aim of selecting patients suitable for Braxon®-assisted PPBR. In recent years, algorithms have been created for helping surgeons in choosing the most appropriate reconstructive technique (77). The preoperative planning for Braxon®-assisted PPBR follows the same procedures as those of the majority of DTI breast reconstruction procedures, and it takes into account the needs of both plastic and oncologic surgeons (78). The type of tumor, localization, dimensions, and breast volume mainly determine the choice of the mastectomy approach (79). Preoperative consultation focuses on scars, planned incision for mastectomy, skin quality and thickness, and patient expectations (80). The best incision is the one that allows an easy mastectomy and a good reconstruction considering previous scars that could affect mastectomy flap viability (81). Skin quality and thickness are usually measured by using the pinch test or digital mammography and magnetic resonance imaging (82). It is widely accepted that a pinch test greater than 1 cm allows prepectoral reconstruction (25, 46).

Preoperative planning continues with accurate measurements that provide the landmarks for drawings and help detect any asymmetries. Attention is focused on the following: the distance from the sternal notch to the nipple, the position of the IMF, breast width, distance from the nipple to the IMF, and distance from the nipple to the midline (83).

To meet the expectations of patients on breast size, a contralateral symmetrization surgery by means of augmentation, breast reduction, and mastopexy can be considered and offered. Preoperative images and patient–doctor dialog are of great help in correctly gauging the condition of patients (84). Patients must be aware of any risk and benefit that each reconstructive technique brings to the table. As for PPBR, the risks include rippling and implant palpability. On the other hand, minimal postoperative pain, avoidance of animation deformity, and maintenance of pectoralis muscle functional integrity are among the most important reported benefits (47, 48, 50, 85). Overall, a shared decision-making process, which must also seek low rates of postoperative complications and a natural esthetic outcome, increases patient satisfaction and postoperative quality of life (30, 43, 45, 86).

For a comprehensive list of the Braxon®-specific articles cited in this paragraph, see Table 2.

## The iBAG study

In the present scenario of a plethora of assorted devices proposed for PPBR, a real limitation is the unavailability of high-quality scientific evidence to support the safety and clinical effectiveness of such appliances. In addition to publications with small cohorts and short follow-ups, most study designs are highly heterogeneous, existing in the form of non-standardized techniques relating to the variability of the biomaterials used, making it difficult to perform a meaningful pooled analysis (18, 53). Such a lack of consistent clinical investigations results in weak data, non-reproducible and unreliable, being of no help in guiding a conscious medical choice (87).

In order to fill this gap, in 2018, the scientific community established a study group strongly oriented toward an extensive and homogeneous multicenter data collection that could obtain significant evidence on outcomes and risk factors related to ADM-assisted PPBR.

**Figure 2:**
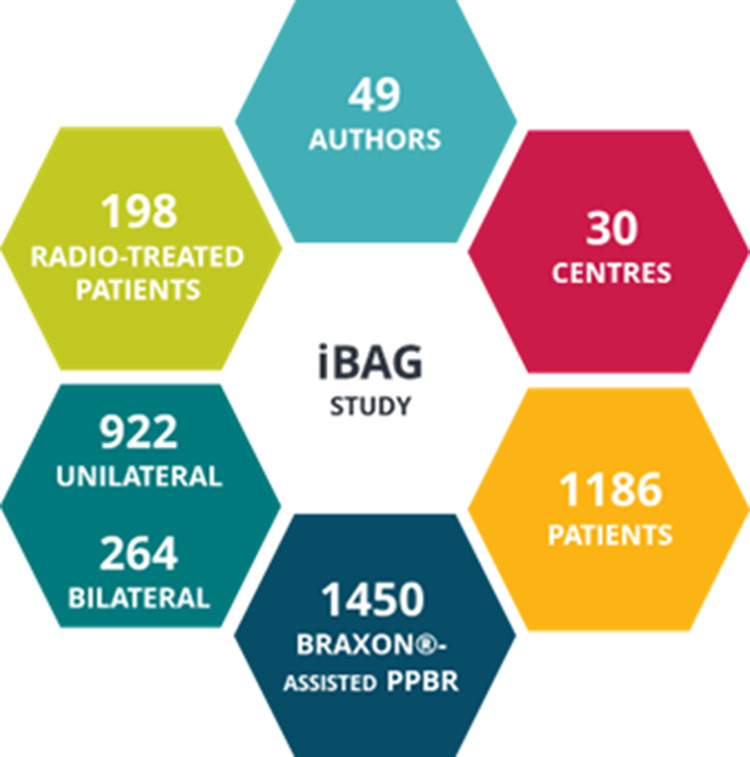
Key points of the iBAG study.

Given its widespread use in European and UK breast centers and the standardized comparable technique, the Braxon® dermal matrix was the ideal choice to create the largest evidence on this procedure, thus constituting the international Braxon Audit Group (iBAG). The iBAG published a study (from now on called “the iBAG study”) characterized as described in Figure 2. Coordinated by the Santa Creu i Sant Pau Hospital of Barcelona (Spain), 30 European centers retrospectively collected data on 1,450 prepectoral Braxon®-wrapped implants, to date the world’s largest data collection on prepectoral implant-based reconstruction. Considering that the only exclusion criterion was patients with less than 3 months of follow-up, clinical–esthetic outcomes reported impressive results: except for seroma at 7%, all recorded postoperative complications had incidences below 5%, with CC incidences among the lowest ever reported of 2.1%. Implant loss was recorded at 6.5%. Interestingly, radio-treated patients (pre- or postoperatively) were also included, enriching the analysis of this patient variable, typically excluded by the selection criteria. Thanks to this large and inclusive cohort, it was possible to reliably link patient-related variables and postoperative complications. This is extremely significant for patient selection criteria and for planning effective and safe reconstruction procedures.

Despite the need for obtaining a higher level of evidence, with this study being a level III study, the data collected from this multicenter audit overall confirm the effectiveness of the technique with low complication rates, especially CC (53).

For a comprehensive list of the Braxon®-specific articles cited in this paragraph, see Table 2.

## Capsular contracture

Breast reconstruction expectations for consistent esthetic outcomes with minimal complications and fast recovery increase continuously for both patients and surgeons (8). In an ideal world, surgical reconstructive outcomes remain stable in time and patients never experience complications that would require reintervention. In real life, however, implant-based breast reconstruction is haunted by capsular contracture, a long-term emerging complication caused by the presence of the breast implant itself (19, 88). The recognition of the synthetic material as “non-self” leads the native tissue to isolate the implant by encapsulating it in a collagen cage: the capsule (21, 89, 90). At the basis of this phenomenon is the foreign body reaction (FBR), characterized by prolonged inflammation at the implant site and copious collagen deposition around the implant for the functioning of myofibroblasts (13, 21). Myofibroblasts also provide the contractile stimulus responsible for contracture (13, 88). Many factors such as implant texture, supposed presence of biofilms, and radiotherapy can lead to this complication by triggering the intrinsic propensity of the capsule to contract (13, 91). Clinically, CC results in cosmetic deformity, pain, and discomfort and is the most common cause of reoperation (92). The need for revision surgery represents an additional burden for women who had already faced the operating theater and took substantial therapies for oncological reasons. As the history of breast reconstruction has shown, covering the prosthesis first by using only muscles, and then by also using ADMs, reduces the risk of CC occurrence in submuscular procedures (18, 23). A Braxon® ADM was specifically conceived to bring breast implants back to the more physiological subcutaneous plane, sparing the pectoralis major muscle, while preventing the formation of a fibrotic periprosthetic capsule (25).

To date, of all the 50+ Braxon® publications, 20 of them have presented data on CC rates, making Braxon® the PPBR-specific biological device with the highest amount of evidence on this topic. For each of these 20 articles, the number of reconstructed breasts, the mean follow-up, and the CC rates (Baker grade III and IV) are reported in Table 4 (Ribuffo et al., 2020 paper is not included because, although they clearly report the CC rate, they consider a mix of devices). It clearly emerges that, apart from one work describing a high rate of Baker grade III CC (but 0% of Baker grade IV CC) (46), capsular contracture overall assesses below the rate of 5%. The weighted mean turns out to be 2.06%. It should be considered that this number is comprehensive for postoperatively radio-treated breasts, known for having a greater risk of CC occurrence. In fact, Polotto et al. confirmed a 10% incidence of CC in patients who had undergone postmastectomy radiotherapy (PMRT) after 28 Braxon® implantations (at a mean follow-up of 24.7 months), while 158 untreated patients (control group) showed a 0.6% CC incidence (a mean follow-up of 21.7 months) (51). A similar outcome was confirmed by the iBAG study on a bigger cohort: of the 200 irradiated breasts, 5.1% developed CC, while only 1.7% of the 1,243 non-irradiated breasts developed CC at a mean follow-up of 22.4 months (53).

**Table 4:** List of the Braxon®-specific published articles in which the CC rates are specified.

Authors	No. breasts	Mean F-UP (months)	CC rates (baker grade III and IV)
Berna et al., 2014	15	14	0
Maruccia et al., 2016	38	12	23.6 (III)–0 (IV)
Berna and Cawthorn, 2017	10	49.2	0
Onesti et al., 2017	64	18	0
Chandarana et al., 2018	71	11.8	1.4
Gardani et al., 2018	51	15.3	0
Chandarana et al., 2019	116	14.4	1.7
Ballesio et al., 2019	19	12	0
Onesti et al., 2020	13	31.2	0
Chandarana et al., 2020	406	11	0.2
Polotto et al., 2020	202	24.3	2
Maruccia et al., 2020	23	23.2	0
Masià et al., 2020	1,450	22.7	2.1
Naemonitou et al., 2020	52	36	0
Spengler et al., 2021	35	9.2	2.9
Mura et al., 2021	111	9	4.5 (III)
Bojanic et al., 2021	1	36	0
Maruccia et al., 2021	46	18.4	0
Gardani et al., 2021	24	19.4	0
Saibene et al., 2022	84	12	3.6

CC, capsular contracture; F-UP, follow-up.

The CC rates are correlated with the number of breasts in the patient cohort and the mean follow-up.

The considerably low capsular contracture rates observed in Braxon®-assisted breast reconstructions confirm the scientific rationale of Braxon® and its effectiveness. Prevention from fibrotic capsule formation is the downstream clinical effect of Braxon® biological activity, which consists of proper integration with the patient's tissues (63). As a result of remodeling, fibroblast colonization, and neovascularization of the collagen cover around the synthetic prosthesis, the capsule that forms at the tissue–implant interface is soft and elastic (63, 93, 94). Such an outcome is indicative of a positive interaction between the matrix and the body and reflects a non-inflammatory state of the breast tissue (27). Prolonged inflammation is strictly linked to fibrosis, which precedes capsular contracture and is a *sine qua non* condition for the onset of capsular contracture. Hence, avoiding fibrosis likely reduces the risk of the patient developing such a complication, also in the long term (21, 95). In fact, no CC was observed 4 years after Braxon® implantation in a small cohort of highly selected patients (54). This result needs to be confirmed on a larger scale; however, the already published data reporting low CC rates at various follow-up times are promising (range 9–36 months).

For a comprehensive list of the Braxon®-specific articles cited in this paragraph, see Table 2.

## Breast-specific matrix: Biocompatibility and adipogenic potential

The infamous FBR at the basis of capsular contracture is caused and boosted by the presence of the breast implant in contact with the breast subcutaneous tissue (19, 21, 27). The reason for the production of such biological effect is to be researched by considering the nature of the foreign material. The word “nature” here is intended as an umbrella term that includes all the intrinsic characteristics of the material: origin, chemical composition, microscopic structure, macro- and micromechanical features, etc. In the case of implant-based breast reconstruction, the prosthesis has a synthetic origin. Despite the compatibility of the prosthesis with the human body, a biological entity - from here the term “biocompatibility” -, their presence triggers the FBR, which is the attempt of the body to expel what is not recognised as part of the body itself (non-self recognition mechanism) (20, 21, 96). Biocompatibility is, hence, the key: it cannot be considered a mere characteristic of the material but rather the interaction of the biological tissue with a specific material and its nature (21). In order to not activate a strong FBR, the material must be recognized as self (19). When this happens, inflammation (always present after a trauma) guiding FBR is modulated and the natural healing process leading to the regeneration of damaged tissues can proceed. Hiding the breast prosthesis under a biological cover such as the pectoralis major muscle helps reduce capsular contracture (and therefore FBR). This indicates that, at the interface with the subcutaneous tissue, switching the material from synthetic to biologic changes and ameliorates the tissue–material interaction (13, 97). The same was also confirmed by placing an ADM sling between the prosthesis and the subcutis in dual-plane reconstruction procedures (23).

In view of sparing the muscle and avoiding all postoperative complications related to its detachment (pain, animation deformity, need for rehabilitation) (47), a complete biological cover of the prosthesis that is able to prevent or reduce FBR to a minimum and promote tissue regeneration is preferable (95). In this scenario, the Braxon® biological matrix has successfully been used for delivering the prosthesis hidden as in a Trojan horse in muscle-sparing breast reconstruction, considerably reducing the risk of CC onset (53). Braxon® is a collagen matrix obtained through a decellularization process that removes all the porcine-specific components from the material, making it biocompatible (25, 53). Its features are designed to meet the requirements of the postmastectomy breast tissue, and they present as follows:

• Collagenic composition. Being collagen among the most conserved proteins in mammals, exogenous collagen is recognized by the body as self. A Braxon® three-dimensional microscopic structure is preserved along the production process, resulting in native collagen organization in the extracellular matrix. This allows efficient matrix repopulation and remodeling by means of fibroblasts and subsequent integration into the subcutaneous tissue (63, 64, 93, 98);
• absence of preservatives and cross-linking agents, not to amplify the inflammatory reaction already present in the tissue because of the surgical insult (25, 93, 99);
• 0.6 mm thickness, which enables matrix integration in timings that follow the collagen *in vivo* turnover, while providing the necessary mechanical requirements of the breast (25); and
• natural collagen fiber structure and resistance. Ethylene oxide sterilization is proven to maintain the collagen's natural features (unlike gamma ray sterilization) (100, 101).

As a dermis-derived collagen matrix, Braxon® reproduces the same structure and biological composition of breast subcutaneous tissues. The biological processes triggered by the matrix do not lead to an absorption of the membrane, but rather to a series of phenomena collectively named “constructive remodeling”: cellular infiltration, physiological degradation, modulation of inflammation, deposition of a new extracellular matrix, and neovascularization (63, 93, 102, 103). The matrix acts as a scaffold promoting host tissue growth (93, 104) and the clinical outcome is tissue reconstitution with matrix transformation into viable self-tissue (63, 105). Evidence of such a result is confirmed by clinical and histological studies: the constructive remodeling process begins immediately after implantation of the Braxon® matrix and continues for the following months, during which the collagen scaffold is progressively integrated into the host tissue. Ultrasound-based investigations corroborate the complete integration of the Braxon® device, according to the timing of the physiological turnover of collagen, which occurs between 6 and 12 months (45, 58, 63, 64).

Among the cell populations present in the breast subcutaneous tissue that work toward its regeneration, the adipose-derived stem cells (ADSCs) provide a crucial contribution. They are the adult stem cells present in the tissue's adipose component, which can differentiate into adipocytes when the breast fat needs to be replenished. Furthermore, they positively influence tissue repair by secretion of anti-inflammatory cytokines and pro-angiogenic factors and by influencing fibroblast proliferation and migration (106–109). A biological matrix able to sustain their activity and differentiation is, therefore, desirable. Additional improvements in Braxon® production technology are aimed at targeting such a biological process. From a recent study conducted at Verona University, *in vitro* and *in vivo* experiments performed on murine models demonstrated that the Braxon® ADM is capable of sustaining ADSC differentiation into adipocytes, with the formation of a well-organized and neovascularized adipose tissue. These results were compared with those obtained with other biological matrices (another dermal matrix and a pericardium). Interestingly, the other biomaterials promoted different responses on the ADSCs: adipocytes derivation occurred later in time or it did not occur at all (64). Braxon® biological and structural similarity with breast subcutaneous tissue, the constructive remodeling it stimulates, and its unique adipogenic features identify Braxon® as the most specific biological scaffold for breast reconstruction. Nowadays, PPBR is performed with various biocompatible materials. However, Braxon® has increased the biocompatibility standard and what can be demanded from a biological scaffold. Such breast tissue specificity makes Braxon® a biomimetic matrix because its peculiar characteristics mimic the breast biological system.

**Figure 3:**
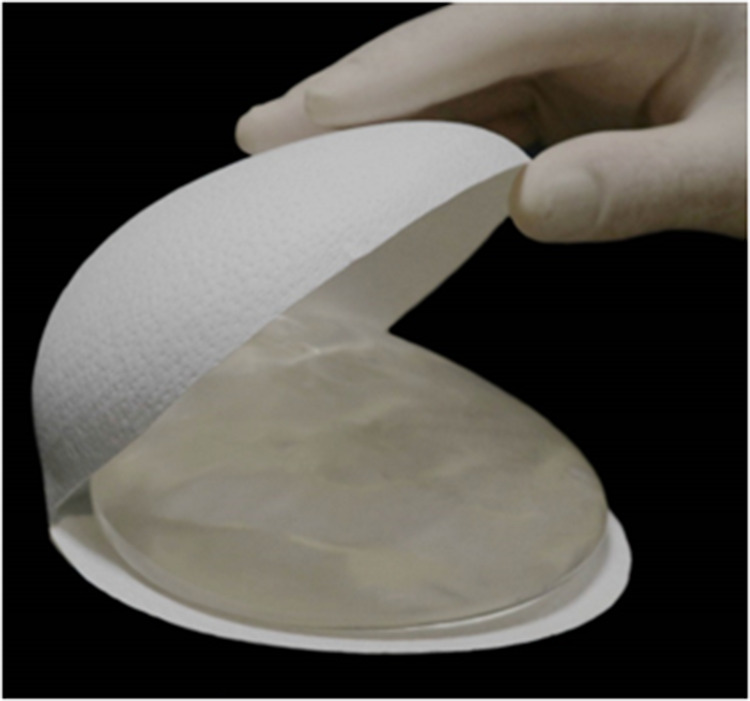
Breast-specific, three-dimensional shaped Braxon^®^ Fast matrix.

The future of breast reconstruction is moving in the direction of developing more and more biomimetic biological matrices. The fourth-generation matrices would mimic not only the biological processes but also the breast three-dimensional shape, to facilitate reconstruction and achieve ever-improving esthetic results stable in time. Very recently, a three-dimensional version of Braxon® has been released: Braxon®*Fast* is a biological matrix that shares the same characteristics of Braxon® (origin, composition, adipogenic properties) but presents a dome-shape anterior face to easily accommodate the implant projection (see Figure 3). The implant remains completely covered by means of a posterior flat side. Such a new 3D conformation speeds up the implant wrapping process, leading to a lower risk of contamination because of less manipulation and less exposure time. In addition, the absence of ribs and folds on the anterior matrix surface after implant wrapping provides an immediate natural reconstructive result (110). Only one article reporting the use of Braxon®*Fast* (110) has been published. We hope that additional publications will be available soon.

For a comprehensive list of the Braxon®-specific articles cited in this paragraph, see Table 2.

## Seroma

When it comes to breast surgery, a discussion on seroma is almost a mandatory step. All breast surgeons are indeed quite familiar with this complication, because seroma formation has always been the most frequent postoperative complication following sole mastectomy, with an incidence rate ranging from 3% to 85% (111).

Although not a life-threatening condition, if not properly prevented or treated, it could represent the first step toward reconstructive failure when a prosthesis is implanted (112). Associated morbidity in the form of prolonged drainage presence is, therefore, not only troublesome to the patient, but can also significantly impact treatment by delaying adjuvant therapy and increasing the risk of infection (113). This results in patient distress, increased office visits, undesirable esthetic outcomes, and—importantly—increased health costs (113).

Data on the incidence of this unpleasant yet common occurrence are good indicators of the quality of a reconstructive device. The literature shows that the Braxon® dermal matrix has been extensively evaluated as well as optimized over time to limit postoperative seroma (25). As a matter of fact, in the first patient series published by Berna et al., it emerges that the very first thicker (0.9 mm) matrix with preservatives was linked to higher seroma incidence compared with the current breast-specific matrix. In fact, clinical evidence–based optimization of ADMs had led to the design of a thinner matrix (0.6 mm) aimed at supporting a physiological and balanced tissue integration and chemical-free composition designed not to exacerbate postintervention inflammation with consequent seroma formation (25).

Recently, Caputo et al. took advantage of the Braxon® standardized wrapping technique to conduct a homogeneous overview on comparable outcomes regarding the incidence of seroma in ADM-assisted PPBR. Upon an analysis of the scientific evidence gathered up to 2021, PPBR with complete ADM-implant coverage shows an overall aggregate incidence of seroma set at 4.9%, which is within the 5% complication threshold of good clinical practice (44).

Incidence of seroma calculated on a large patients cohort such as that of the iBAG study is confirmed to be in line with the scientific literature standards, although it is set slighly higher (7%). This result, which is just above the aforementioned threshold, must, however, be contextualized with the inclusion of non-ideal patients with comorbidities (53).

Overall, clinical experience shows low incidences of seroma, mostly in line with good clinical practice. According to expert users of Braxon®, however, fluid accumulation can be prevented by (i) minimizing intra- and postoperative inflammation (44, 114, 115), (ii) obliterating postmastectomy dead spaces (44, 111, 113), and (iii) ensuring intimate tissue contact and mechanical stability of the ADM to facilitate its integration (33, 44).

For a comprehensive list of the Braxon®-specific articles cited in this paragraph, see Table 2.

## Radiotherapy

Together with seroma and capsular contracture, the clinical–esthetic effect of radiation therapy on breast surgery is a current hot topic of great interest worldwide. Radiotherapic treatment has indeed become a widespread and well-established tool in breast cancer management because it reduces loco-regional oncological recurrence (51). This, however, has thrown up many questions about its policy of use in conjunction with breast reconstruction, as it is a widely known risk factor causing complications and leading to increased fibrosis (91, 95). For this reason, the last 10 years have seen strict guidelines on considering RT as an obstacle to PPBR (49). Nevertheless, while neoadjuvant RT is preoperatively administered, it cannot always be predicted whether PMRT will be needed. Over time, this has led to the collection of many clinical insights even on patients not strictly suitable for PPBR, allowing a study of reconstructive material behavior in this specific setting (53).

Braxon® is one of the few breast-reconstructive devices for which clinical outcomes in conjunction with RT are available (27). At the beginning of 2020, Polotto et al. published the results of a retrospective analysis on Braxon®-assisted PPBRs in the setting of PMRT. Radio-treated patient outcomes were compared with those in a non-irradiated control group, with the specific aim of evaluating whether the complete implant wrapping technique can be safely recommended in the event of PMRT. Apart from a higher rate of CC (0.6% for non-irradiated patients vs. 10.7% for radio-treated patients), they found no significant differences in complication and failure rates between the two groups, thus even assuming a protective role for ADMs from the effects of PMRT (51).

Through a univariate analysis, the iBAG study goes even further investigating the effect not only of PMRT, but also of preoperatively administered radiotherapy. In a cohort of 198 radio-treated patients (including 45 preoperatively treated patients and 159 postoperatively treated patients), it was found that there were significantly higher incidences of seroma, capsular contracture, and implant loss in the radio-treated group. High incidences of CC were mostly associated with PMRT, with an incidence of 6.3% compared with 1.7% in non-irradiated patients. Radiotherapy had no other statistically significant link to other complications (53).

While the iBAG analysis confirms the results obtained by Polotto et al., the conclusions of a very recent 2022 study differ. In 84 Braxon®-assisted PPBRs, outcomes reported for 18 irradiated breasts (22.2% preoperative, 72.2% postoperative, and 5.5% both treatments) reveal a statistically significant association between RT and postoperative complications only in the case of infections (6.1% vs. 22.2% non-irradiated and irradiated patients, respectively) and implant loss (6.1% vs. 33.3% non-irradiated and irradiated patients, respectively). These findings will certainly have to be confirmed by larger and more significant cohorts but could help design patient-specific antibiotic protocols minimizing infection occurrence. Furthermore, when considering RT, an increased rate of CC was recorded in irradiated patients, although not significant, likely due to the small sample size (27).

It should be noted that the above-mentioned studies do not distinguish between early and late complications in PMRT settings. In this scenario, because RT should ideally be administered not earlier than 3 months post reconstruction, some of the complications that most commonly occur in the early postoperative period (<3 months) and considered linked to PMRT may not be really related to it and should, therefore, not be reported as such.

In addition to the three publications mentioned here, other scientific evidence reports radio-treated patients in Braxon®-assisted cohorts, but without specific and separate data analyses that could allow significant observations to be made (26, 33, 57, 65). The results collected so far are still limited by medium-small cohorts and medium follow-ups, but they can serve as an excellent starting point for future randomized prospective studies.

Given these relatively early experiences, RT is no longer considered an absolute contraindication to PPBR (51, 53).

For a comprehensive list of the Braxon®-specific articles cited in this paragraph, see Table 2.

## Braxon® in revision surgery

Direct-to-implant PPBR with Braxon® has been widely reported with satisfactory results (25, 35, 48, 51, 53, 63, 72). Less described, however still successful, is the use of such a matrix in cases of revision surgery for the treatment of a variety of postoperative complications. In the very first cohort of Braxon® patients, two of them were undergoing revision surgery for dancing breast syndrome and a double capsular contracture, respectively, which were causing pain and poor esthetic results. After pocket conversion and prepectoral implant placement, no pain and no major complications were reported at a mean follow-up of 14 months (25). Mangialardi et al. reported this type of intervention involving pocket conversion and prepectoral Braxon®-wrapped implant placement on a wider cohort of 19 patients. These patients presented with submuscular prosthesis and suffered from functional and esthetic complications such as severe animation deformity, implant malposition, alteration of shape, dysfunctional chronic chest pain, and infection-caused implant loss. The authors, in addition, identified as suitable those patients who were in need of a subpectoral tissue expander substitution after nipple-sparing mastectomy, had previous contralateral breast reconstruction with autologous tissue, and could not face a second free or local flap due to clinical or psychological reasons (one patient). A more detailed pinch test was used as an additional patient selection criterion: with >3 cm of pinched tissue at the upper pole and >1 cm at the lower pole, the patient was considered a good candidate; with a measurement in between 1.5 and 3 cm at the upper pole and >1 cm at the lower pole, the patient needed to receive one or more preparatory fat grafts before surgery; with <1.5 cm at the upper pole, the patient was excluded. At a mean follow-up of 14.2 months, only one case of a patient with seroma, conservatively treated, was observed, none of the complications that led to revision in the first place occurred, and a high level of satisfaction with the surgical outcome was reported by patients (43). Braxon®-assisted revision surgery has also been performed for treating capsular contracture and preventing its recurrence. In a case–control study, 42 patients with submuscular implants underwent complete anterior capsulotomy and received the bare prosthesis (control group) or the ADM-wrapped prosthesis (ADM group), with no plane change. Capsular contracture recurrence was lower in the ADM group; however, this result did not show any statistical significance. Of note, in the patients' cohort, some of them who underwent RT were present, thus explaining the onset of capsular contracture (30). It is worth noting that cases of Braxon®-assisted revision exist in the iBAG study (41 out of 1,450). Their outcomes are, anyway, described in the unique result dataset (53).

The safety and efficacy of Braxon® application in revision surgery extends to complex clinical cases [silicone migration and fistulization into the pleural space after breast implant rupture (66)] and esthetic breast surgery. In the case report published by Bojanic et al., Braxon® implant coverage allowed a patient, who had multiple breast surgeries and implant-related complications, to eliminate the pain she was experiencing, in order to resolve prosthesis hypermobilization with lateral displacement and to amend the unnatural nature of the breasts. At 3 years post revision, the result was still optimal, with no signs of capsular contracture and implant mispositioning (42).

When patients are properly selected, reported available data are encouraging with optimal and stable results, especially considering that those undergoing revision surgery are more prone to complications (53, 116). However, data are still limited, and further studies are needed to determine whether the use of ADMs is significant in this definite surgical specialty.

For a comprehensive list of the Braxon®-specific articles cited in this paragraph, see Table 2.

## Cost-effectiveness

With the increasing number of available materials and techniques, it has become progressively difficult to evaluate the cost-effectiveness of reconstructive strategies (4). This represent a huge limitation for clinical development as long as it remains anchored to traditional submuscular techniques which, however, can be even more expensive than making use of the latest technologies available (68, 69). Overall, the literature still lacks reproducible demonstrations regarding cost–benefit comparisons between new ADM-assisted techniques and conventional two-stage submuscular approaches (68, 69). Therefore, here, we report an organized collection of Braxon® cost–benefit analyses of the latest scientific evidence.

The cost-effectiveness of the device is first verified by the health technology assessment of Parma University Hospital (67). According to hospital sources, ADM-assisted one-step surgeries allow a cost reduction for the public health system of 15%, due to the avoidance of the unnecessary second surgery (67). The Braxon® literature, however, also includes several specific cost–benefit articles that may be helpful (68, 69). In particular, Cattelani et al. evaluate the cost-effectiveness of the one-step Braxon® technique, reporting a solid clinical-economic advantage over one-step and two-step submuscular procedures. According to this original study, the use of a two-stage procedure may result in acceptable esthetic outcomes, but besides demanding important functional toll, it almost doubles the direct costs because of the two surgical steps, and it causes a far longer period of physical and emotional disability for the patient compared with single-stage procedures (68, 69). Even submuscular DTI procedures are no more advantageous than the prepectoral wrapping technique, due to higher symmetrization costs generated by the poor and less natural results of the submuscular reconstruction procedure.

Muscle preservation, in addition, resulted in a lower pain intensity and a lower consumption of analgesics—also confirmed by a subsequent publication by Caputo et al. and Vidya et al. (48, 72). As a consequence, costs were further lowered, whether they were chargeable to the patient or to a publicly funded healthcare system.

For a comprehensive list of the Braxon®-specific articles cited in this paragraph, see Table 2.

## Quality of life

The type of surgical technique selected for patients impacts their quality of life. PPBR is less invasive and causes less pain compared with other reconstructive strategies (48, 72). Various questionnaires (BreastQ, EORTC QLQ-C30, and QLQ-BR23) administered to assess patients' global status after surgery have shown high scores, indicating excellent results in the investigated domains also in cohorts of elderly patients and of high BMI patients (35, 37, 45, 71). When the same investigation was performed by comparing the results of patients with subpectoral implants or expanders and with Braxon®-covered prepectoral implants, these were higher in the PPBR cohort, indicating a better preservation of the quality of life for these patients (46, 68, 72). Muscle involvement in breast reconstruction has functional consequences on upper limb movement and hence on the patient's daily life. Compared with subpectoral breast reconstruction, PPBR allows us to retain a greater range of motion of the glenohumeral joint on the operated side. In fact, with the muscle-sparing technique, flexion, internal and external rotation, and abduction are better retained at 30 days from surgery. In addition, PPBR patients are less likely to require an individual rehabilitation program to achieve complete functional limb recovery (72). With less upper limb functional impairment, patients return faster to their daily lives and routine (68).

Long-term quality-of-life investigations performed on big cohorts of patients are needed to confirm the results. However, the indirect benefits of Braxon®-assisted PPBR are evident, and thus, offering such reconstructive possibility to patients means offering them the best social wellness achievable in a setting of breast cancer therapy.

For a comprehensive list of the Braxon®-specific articles cited in this paragraph, see Table 2.

## Discussion

Immediate implant-based breast reconstruction following mastectomy is oncologically safe and improves patients' psychosocial health (2, 87). A variety of techniques for breast reconstruction have been developed during the past 50 years to lessen the negative influence of mastectomy on patients' quality of life (4, 72). Among the several methods, the prepectoral approach is currently one of the most promising techniques in this surgical field, regarded by many as the new reconstructive gold standard (27). Although a much-praised and widespread technique nowadays, till 10 years ago, prepectoral reconstruction was deemed a risky and hopeless approach: a muscle sacrifice was considered inevitable, and breast psychophysical recovery was at the expense of pain and anatomical function (18, 25, 72).

### Why then do we perform prepectoral reconstruction surgeries today?

ADMs are the answer: What is different today is that we have a biomaterial capable of overcoming synthetic-induced fibrosis (22, 25, 117). After mastectomy and synthetic inert expander/implant/mesh insertion, the tissue in contact with the device is essentially a raw wound bed that heals by secondary intention, that is, scar and capsule formation (27, 81). The organism, in fact, not recognizing the structure or composition of the implanted material, starts an inflammatory foreign body reaction that isolates it through a fibrotic envelope (21), also referred to as a “stiff and rigid cage” (89). According to Saibene et al., the literature reports cumulative CC incidences ranging between 6% and 18% with synthetic implantation. Even worse CC rates (16%–60% more) occur with reconstruction procedures in conjunction with RT treatments (27).

If at first the fibrotic foreign body manifestations were masked with a submuscular prosthetic positioning, more recently, the evolution of biological scaffolds such as ADMs is leading breast reconstruction toward higher standards of biocompatibility (21, 27, 96). According to Liu et al., in fact, the various clinical applications of biomaterials have long remained hindered because of the intrinsic inertia of synthetic materials (75). Contrary to the latter, ADMs are bioactive materials that demonstrate a superior biocompatibility, as they retain many of the native proteins and three-dimensional biological structures necessary to guide the patient's cell adhesion and migration, modulating inflammation toward regenerative healing (19, 104). Although the underlying mechanisms have not yet been fully understood, numerous clinical–histological studies have been demonstrating how the ADM is associated with reduced inflammation and capsular contracture (22–24, 118). In this regard, as Brown, Garner, and Young demonstrated that the capacity of a skin graft to inhibit wound contraction is directly proportional to the amount of structurally intact dermal collagen present in the graft, Tevlin et al. suggest that the same could happen with ADMs: “Incorporation of ADM in the reconstruction allows the internal breast wound to be ‘grafted’, thus potentially halting the unfavorable sequelae of contracture formation, analogous to skin grafting an open wound” (13).

The complete wrapping of a breast prosthesis with a decellularized collagen sheet made it possible to exploit the body’s regenerative potential in the breast (19, 22), colonizing the prepectoral space in a feasible way (25). Braxon® is the very first preshaped and patented ADM to continuously wrap the synthetic prosthesis, thus exerting a regenerative action on the entire surface of the implant interface with patient tissues (25).

Whether the ADM surface should be fenestrated or non-fenestrated (continuous) is a debated topic. Recently, different fenestrated biological matrices have become available in the PPBR market scenario. Nonetheless, given the rationale behind the use of these devices in the prepectoral space, it is likely that fenestrated ADMs could have a reduced regenerative potential, not providing protection from fibrotic healing right within the holes of these collagen sheets. Studies currently available on PPBR and fenestrated ADMs seem to confirm this hypothesis. Compared with 2.1% CC incidences reported in the iBAG study (53) and the weighted mean of 2.06% in this study, which is calculated with the Braxon® continuous collagen sheet, fenestrated-ADM-assisted PPBRs outline higher CC incidences: out of 71 breasts, Scheflan et al. found an incidence rate of 8.5% of CC (119), while in the same year, Fin et al. recorded 6% (120). More recently, Wazir et al. reported a 12.5% incidence rate (121).

Alongside fenestration, the derivation source of the biological matrix would seem to influence the clinical result as well (93): while dermis-derived materials have been extensively implanted and studied in the breast (93, 98, 122), a pericardium-derived scaffold gave a suboptimal result in breast reconstruction (123). Moreover, as a result of a literature research, no studies with significant samples and follow-ups regarding the incidence of CC with pericardium materials were found.

As part of what can historically be identified as a third generation of breast ADM, Braxon® today appears to be finely engineered to be biomimetic with breast tissue (27, 64, 124). Nonetheless, this was clearly possible through experience gained with the previous generation, mostly ADMs imported from abdomen surgeries (15). The abdomen is an extremely different site from the breast, being subjected to different pressures and cellular environment. Not being initially designed and indicated for the breast implant site, the morphological and mechanical properties of these previous ADMs were not refined, and many postoperative complications emerged, such as the onset of seroma (17).

Although seroma is the most reported postmastectomy complication (111), fluid accumulation has henceforth been indelibly linked to the use of ADMs (44). Chemical additives initially present on the first generation of breast ADMs, adapted from those used in abdominal surgery, are likely involved in the ADMs bad name. In fact, preservatives amplify the tissue inflammatory response and exacerbate the formation of seroma, leading to think that the ADM per se causes such complication. (25, 125, 126). This was also testified by Berna et al. comparing the Braxon® seroma results with those of a previous additive version of Braxon® (25). However, this hypothesis would support an inflammatory origin of the seroma complication, which, even today, is often regarded as etiologically orphaned (44). A reference that helps to clarify this issue is a publication dated 2015, where it was investigated whether the presence of ADMs influenced the daily postoperative serum collection pattern (127). A comparison of the data on serum collection between submuscularly reconstructed patients and only mastectomized patients showed that the trends were very similar, and the drainage patterns followed the development of the physiological inflammatory response. Even in the specific prepectoral context, seroma is not correlated with ADMs (44). A homogeneous analysis by Caputo et al. on the Braxon® matrix outlines an aggregate incidence of seroma of 4.9%, an extremely significant result compared with 85% reported in the literature for mastectomy alone or incidences reaching up to 65% with synthetic meshes (44). This confirms that the technical characteristics of the implanted material inevitably influence the clinical result (93), be it biological or synthetic. Together with the technical specificity of the material, however, the clinical evidence analyzed here suggests seroma as a side effect of surgery rather than a complication of breast reconstruction (44, 111). In particular, there are three elements that contribute predominantly to fluid accumulation, namely (i) destruction of the lymphatic vessels, (ii) inflammatory fluids, and (iii) presence of dead spaces (44). As a matter of fact, after mastectomy, the mammary site is depleted of the lymphatic system, which no longer adequately drains the liquids formed as a result of the mastectomy-dependent inflammation. In addition, there are inevitable dead spaces (111, 113), derived from mastectomy tissue detachment. Preventive measures must, therefore, be taken to limit each of these factors to a minimum, starting with gentle surgeries that do not excessively stress the tissues (low electrocautery voltage) (114, 115), no povidone-iodine in the pocket (128, 129) to the use of materials that do not exacerbate the inflammatory process, such as synthetics or matrices not optimized for the implantation site (89). As for the reduction of dead spaces, several authors suggest separate pockets for mastectomy and axillary dissection, as well as the application of sutures between the matrix and the subcutaneous layer, which have proved to decrease fluid accumulation (33, 44).

Literature on the breast is doubtless moving toward gathering higher-quality scientific evidence, and the Braxon® acellular matrix has been an excellent starting point for this revolutionary paradigm. Alongside the previously discussed iBAG study, this device has been the subject of several Italian (34) and European (26, 32, 59, 65) multicenter studies, as well as countless monocentric experiences (27, 30, 43, 45, 57, 61, 62). In all cases, clinical practice shows results in line with traditional submuscular complications (55, 130), if not extremely improved (34, 68, 69, 72). Except for sporadic minor esthetic problems such as rippling (50), literature on Braxon-assisted PPBRs report effective clinical – esthetic outcomes (53) with even a reduction in health care costs when compared with traditional submuscular reconstructions (67–70).

Beyond the issues already discussed exhaustively here, this porcine-derived matrix collects extremely heterogeneous clinical experiences such as implants in association with polyurethane prostheses (39), revision treatments (30, 42, 43, 66), comparisons with other collagen-based scaffolds on the market (60), and also several publications that are now proposed to expand the selection criteria for elderly patients (46, 71), skin-reducing surgeries (35, 37, 38, 131), as well as risk-reducing operations (61).

However, one of the most valuable insights that this device has gathered is the evidence in the radiotherapy setting. Having proved to be a safe scaffold producing stable results over time (53), several research groups have studied its clinical outcomes even outside the stringent PPBR guidelines on RT. In other words, a slight increase in the incidence of capsular contracture was found, but without any worsening in the overall and final reconstructive outcome (27, 51, 53). According to the literature, prepectoral irradiation would have more lenient side effects than submuscular irradiation (23, 27, 95, 132), which entails the risk of patients incurring muscle fibrosis with consequent muscle shortening and tightening, elevating any underlying device as the implant pocket starts to contract (27). Sigalove and Saibene and colleagues firmly argue that the reconstruction outcome is strongly influenced by the reconstructive plan and by the material used, when RT is administered. Based on the proven ADM regenerative potential and on early published evidence, a possible protective role for ADMs from the effects of RT is, therefore, hypothesized (27, 95).

The premises seem promising, but similar hypotheses and clinical results must still be confirmed with further studies, in order to ensure a safe clinical practice.

In the current implant-based PPBR scenario, the run through different biomaterials is producing massive non-comparable outcomes that are disorienting consistent medical guidelines. To fill this gap, we recollected and reviewed all Braxon®-published scientific literature: not only the biological matrix with the highest number of scientific studies in PPBR, but also the only ADM with a specific patented design that allows a standardized wrapping technique (26, 53), reducing the number of variables and thus giving a more coherent and homogeneous perspective of this area of surgical specialization (44).

However, this publication has some limitations. Because this was a narrative review, a comprehensive analysis on Braxon® and Braxon®*Fast* accessible data was not performed, and the presented evidence cannot comment on PPBR results gathered from other biomaterials currently available. In this regard, there are no meaningful available data on comparative outcomes with various ADMs and this is unlikely to be available in the near future. Furthermore, the follow-up of the different studies is variable in nature, and therefore, more reliable long-term data will be needed for determining capsular contracture rates.

## Conclusion

This study concludes that advanced breast reconstruction techniques require the use of improved biomaterials: a prothesis wrap-around with dermis-derived material enabled prepectoral breast reconstruction, thus favorably affecting the quality of life of oncological patients. Material structure and composition deeply influenced the medical implications of the procedures. As an evolution of previous generations of ADMs, the Braxon® dermal matrix is the medical device with the largest collection of clinical evidence in PPBR, outlining a safety profile with stable and reproducible results.

The standardized Braxon® technique, together with a strong scientific rationale, allows a homogeneous and meaningful analysis of the prepectoral approach, from patient selection and operative technique, to cost-effectiveness, to the prevention and management of complications.

Strict selection criteria need to be suggested to the new ADM user: as the learning curve progresses, broader criteria can be applied to yield results in line with good clinical practice.
